# Vitamin D deficiency in community-acquired pneumonia: low levels of 1,25(OH)2 D are associated with disease severity

**DOI:** 10.1186/1465-9921-15-53

**Published:** 2014-04-27

**Authors:** Mathias W Pletz, Christoph Terkamp, Ulrike Schumacher, Gernot Rohde, Hartwig Schütte, Tobias Welte, Robert Bals

**Affiliations:** 1 Center for Infectious Diseases and Infection Control, Jena University Hospital, Erlanger Allee 101, 07740 Jena, Germany; 2 Department of Gastroenterology and Hepatology, Hannover Medical School, Hannover, Germany; 3 Center for Clinical Studies, Jena University Hospital, Jena, Germany; 4 Department of Respiratory Medicine, Maastricht University Medical Centre (MUMC+), Maastricht, the Netherlands; 5 Department of Respiratory Medicine, Charite, Berlin, Germany; 6 Department of Respiratory Medicine, Hannover Medical School, Hannover, Germany; 7 Department of Internal Medicine V – Pneumology, Medical Centre of the Saarland University, Homburg, Germany; 8 CAPNETZ Foundation, Hannover, Germany; 9 Center for Sepsis Control and Care, Jena University Hospital, Jena, Germany

**Keywords:** Bacterial infection, Pneumonia, Viral infection

## Abstract

**Objectives:**

We aimed to explore the association between vitamin D levels and the severity, mortality and microbiological etiology of community-acquired pneumonia.

**Methods:**

Vitamin D levels (both, the reservoir form 25-OH and the activated form 1,25-OH2) of 300 randomly selected patients with community-acquired pneumonia due to pre-specified pathogens included in the German competence network (CAPNETZ) study were measured. Prior to statistical analysis, values of 25-OH and 1,25-OH2 were power-transformed to achieve parametric distribution. All further analyses were performed with seasonally and age adjusted values.

**Results:**

There was only a modest (Spearman Coefficient 0.38) positive correlation between 25-OH and 1,25-OH2. For 1,25-OH2 but not 25-OH, the general linear model revealed a significant inverse correlation between serum concentration and CURB score (p = 0.011). Liver and respiratory co-morbidity were associated with significantly lower 25-OH values and renal co-morbidity with significantly lower 1,25-OH2 values. No significant differences of 1,25-OH2 or 25-OH between different pathogens (influenza virus, Legionella spp., Streptococcus pneumoniae) were detected.

**Conclusion:**

For 1,25-OH2, we found a significant and independent (controlled for age, season and pathogen) negative correlation to pneumonia severity. Therefore, supplementation of non-activated vitamin D to protect from pneumonia may be non-sufficient in patients that have a decreased capacity to hydroxylate 25-OH to 1,25-OH2.

## Introduction

There is growing evidence that the effects of vitamin D reach far beyond calcium and bone metabolism. In recent years several immune modulatory effects of vitamin D have been described.

Biological activation of vitamin D requires a stepwise hydroxylation to first vitamin D 25-OH by the 25-hydroxylase (CYP27A1) in the liver. The second hydroxylation is catalyzed by 1α-hydroxylase (CYP27B1) in the kidney and reveals finally the biologically active form of vitamin D: vitamin D 1,25-OH2. Whereas 1,25-OH2 exhibits a short half live of 4-6 h, the much less active precursor 25-OH can be considered as a reservoir with a substantially increased half live of 3 weeks. Recently, it has been recognized that besides the kidney, leukocytes contain 1α-hydroxylase and can therefore activate 25-OH [1].

No common definition exists for adequate vitamin D status measured as 25-OH serum concentrations. A recent guideline has been suggested that vitamin D deficiency be defined as 25-OH below 20 ng/ml, insufficiency as 21–29 ng/ml, and sufficiency as 30–100 ng/ml [2]. According to this guideline, serum 1,25-OH2 does not reflect vitamin D reserves and is frequently either normal or even elevated in those with vitamin D deficiency [2].

There is an increasing number of in vitro, ex vivo and animal studies describing the effects of Vitamin D on the innate immune response as well as on the B and T cell response with sometimes contradictory results (for review see [3].

In summary, 1,25-OH2 seems to facilitate direct antimicrobial effects of the innate immune system and to attenuate an (over-)whelming inflammatory response by modulating the crosstalk between cells of the innate immune system and T cells.

Prospective studies investigating a possible protective effect of vitamin D alimentation against respiratory infections revealed also conflicting results [4]. In fact, there are only 2 high quality vitamin D alimentation studies that showed an improvement of a clinically relevant endpoint: An randomized controlled trial (RCT) by Nursyam et al. found an increased sputum conversion rate in 67 Indonesian adult patients with tuberculosis [5] and Urashima et al. reported a significant reduction of Influenza A infections (relative risk 0.58; 95% CI 0.34, 0.99) in 187 Japanese children [6]. Recently, a systematic review on 39 studies addressing the value of vitamin D on prevention of respiratory tract infections (4 cross-sectional studies, 8 case–control studies, 13 cohort studies and 14 clinical trials) satisfying review eligibility criteria was published. According to the authors, results from RCTs were conflicting and they suggested to conduct studies in populations with a high prevalence of vitamin D deficiency at baseline, using doses sufficient to induce sustained elevation of serum 25-OH to detect an effect [4]. All these studies used vitamin D and none of its metabolites for supplementation.

Our study aimed to identify associations between disease severity and/or specific respiratory pathogens in community-acquired pneumonia (CAP) and the levels of vitamin D reservoir form 25-OH and its active form, 1,25-OH2. In contrast to other studies, that assessed only D 25-OH (the vitamin D “reservoir”); we measured the serum concentration of both, 25-OH and 1,25-OH2 (the “active” metabolite) and we used transformed values to control for the impact of age and season.

## Methods

### Design and patient population

This was a cross sectional study analysing vitamin D levels of 300 randomly selected patients enrolled into the large prospective CAPNETZ cohort study. The inclusion criteria for the CAPNETZ study were age > =18 years, the presence of a new infiltrate on chest radiography, and at least 1 of the following criteria: history of fever (temperature > =38.3°C), cough, production of purulent sputum, or focal chest signs on auscultation. Patients who had been hospitalized during the 28 days preceding the study patients with severe immunosuppression or active tuberculosis were excluded. The study was approved by the ethical review board of each participating clinical centre, and all patients included gave informed consent. Detailed information on CAPNETZ methodology is provided elsewhere [7]. Follow up by a phone call (patient or next of kind or family physician) was conducted 30 +/- 2 days and 180 days after enrolment.

### Sample selection

Samples of patients with confirmed pneumococcal pneumonia (n = 100), confirmed influenza pneumonia (n = 50), confirmed legionella pneumonia (n = 49), and without any pathogen detected (n = 101) were randomly selected by using the random sample selection function of SPSS 20.0. Samples were derived from patients due to selected respiratory pathogens supposed to induce different immunological responses. All samples were taken at the time of CAP-diagnosis.

### Data collection

In this prospective cohort study, all demographic, clinical and diagnostic data of the patients were recorded using standardized web-based data acquisition. The study period comprised 79 months starting on 1st June 2002 and ending 31st December 2008.

Pneumococcal vaccination status was considered positive, if patients had received pneumococcal vaccine within the last 5 years prior enrolment.

### Processing of samples

All available respiratory specimens and blood cultures were immediately processed in the local microbiology laboratories of the participating clinical centers. Gram staining and culture were performed for all respiratory samples. Undiluted PBS and BAL fluid samples were also cultured on charcoal-yeast extract agar if *Legionella spp.* was suspected. Urine was tested for the presence of *S. pneumoniae* and *Legionella spp.* antigens using the Binax Now test and Legionella Now test (Binax Inc), respectively. Virus identification and further subtyping was carried out as described previously [8].

25-OH was measured in serums samples obtained at enrolment by the LIAISON® 25 OH Vitamin D TOTAL chemiluminescent immunoassay from DiaSorin, Germany according to the manufacturer`s protocol. 1,25-OH was measured in serums samples obtained at enrolment by the 1,25-Dihydroxy Vitamin D EIA kit from IDS, Germany according to the manufacturer`s protocol. This is a complete assay system for the purification of 1,25-OH2 in human serum by immunoextraction followed by quantitation by enzyme immunoassay.

### Definitions

*S. pneumoniae* was considered as pathogen when (i) isolated from blood cultures or pleural fluid cultures or (ii) in the presence of a good quality sputum revealing > 25 polymorphonuclear cells and < 10 epithelial cells per power field (total magnification × 100) and predominant growth in culture of sputum (≥10^6^ cfu) or BAL (≥ 10^4^ cfu/mL) or (iii) when the antigen was detected in urine.

The diagnosis of *Legionella* pneumonia was considered definite if the patient met one of the following criteria: (1) isolation of *Legionella* species from the culture of a respiratory sample; (2) detection by PCR; or (3) detection by urinary antigen testing.

Patients with PCR - positive influenza respiratory samples without a secondary bacterial or viral pathogen detected were defined as influenza pneumonia, therefore bacterial superinfections were excluded.

Vitamin D deficiency was defined as a D 25-OH below 20 ng/ml, insufficiency as 21–29 ng/ml, and sufficiency as 30–100 ng/ml [2]. For D 1,25-OH2 deficiency, there is no established cut off [2]

### Statistics

Prior to statistical analysis data of 25-OH and 1,25-OH2 were power-transformed to achieve parametric distribution. Due to the well-known seasonal variation, we first examined the influence of the month of blood sample date on vitamin D levels. All further analyses have been performed with seasonally adjusted (monthly mean) values of both, 25-OH and 1,25-OH2.

Influence of possible confounders like age, duration of respiratory symptoms until blood drawing, presence of renal comorbidity, pneumonia severity (CURB) and inflammation markers have been tested by general linear models (GLM). For age as a general confounder was adjusted as well in all subsequent models.

In a final step, 25-OH and 1,25-OH2 levels in the subgroups formed by the pathogens were compared by means of general linear models with pathogen as factor.

P-values (p) and respective test statistics (degrees of freedom, F_x,y_) are displayed.

There was only one single case of missing data for Vitamin D values. This one was handled as such and not replaced by estimates. Patients with missing data in the covariates were excluded from the respective analysis.

All analyses were performed with the SAS software package, version 9.3.

## Results

### Descriptive statistics

Patients’ characteristics and comparison between the four groups (no pathogen detected, influenza, legionella and *S.pneumoniae*) are displayed in Table 1. Except for hepatic co-morbidities (highest proportion in patients with pneumococcal pneumonia), sex (high proportion of males in patients with legionella) and D 25-OH (lowest transformed values in patients with influenza) and CRP levels (lowest values in patients with influenza), there were no statistically significant differences. Considering the non-transformed values of 25-OH, 82% of the cohort exhibited vitamin D deficiency.

**Table 1:** Patient’s characteristics

**Parameter**	**No pathogen detected (n = 101)**		**Influenza (n = 50)**		**Legionella (n = 49)**		**Streptococcus pneumonia (n = 100)**		**ANOVA resp. Fishers exact test**	
		**n**		**n**		**n**		**n**	**Test statistic**	**p value**
Age (mean, SD)	59.43	15.9	60.13	17.8	62.65	16.7	57.41	17.0	F_3,296_ = 1.12	0.3403
Sex (male % n)*	61.4%	62	52.0%	26	71.4%	35	49.0%	49		0.0437
Active smoker	40.6%	41	38.0%	19	36.7%	18	43.0%	43		0.8855
Heart insufficiency	11.9%	12	12.0%	6	20.4%	10	11.1%	11		0.4435
Cerebrovascular co-morbidity	5.1%	5	10.0%	5	2.0%	1	7.0%	7		0.3649
Diabetes mellitus	13.9%	14	10.0%	5	22.4%	11	13.0%	13		0.3454
Respiratory co-morbidity	33.0%	33	30.0%	15	38.8%	19	32.0%	32		0.8059
Hepatic co-morbidity*	0.0%	0	2.0%	1	6.1%	3	7.0%	7		0.0167
Renal co-morbidity	5.0%	5	6.0%	3	12.2%	6	6.0%	6		0.4034
Vaccination with the 23 PPV	14.3%	14	10.0%	5	14.3%	7	6.1%	6		0.1158
Vaccination with the TIV	38.4%	38	32.0%	16	37.5%	18	23.5%	23		
From nursing home	3.0%	3	2.0%	1	0.0%	0	4.0%	4		
CURB-index										0.2829
0	56.0%	42	50.0%	21	55.6%	20	38.7%	29		
1	32.0%	24	38.1%	16	33.3%	12	52.0%	39		
2	10.7%	8	11.9%	5	8.3%	3	9.3%	7		
3	1.3%	1	0.0%	0	2.8%	1	0.0%	0		
Hospitalization	58.2%	57	65.3%	32	75.5%	37	71.1%	69		0.1259
180 day mortality	7.1%	7	4.1%	2	6.4%	3	1.0%	1		0.1154
30 day mortality	1.0%	1	2.1%	1	2.1%	1	0.0%	0		
Deficient/insufficient/sufficient	74.3/16.8/8.9%	75/17/9	92.0/2.0/6.0%	46/1/3	77.6/22.4/0%	38/11/0	82.3/12.7/5.0%	87/9/3		
D3 25 OH [ng/ml] (mean ± SD)**	16.2 ± 9.0		11.3 ± 9.7		14.1 ± 7.1		12.9 ± 7.4		F_3,295_ = 6.66	0.0002
D3 1,25 OH2 [pg/ml] (mean ± SD)***	51.5 ± 24.6		54.5 ± 37.6		52.4 ± 38.4		42.4 ± 23.0		F_3,295_ = 2.43	0.0651
CRP [mg/l] (mean ± SD)*	105.2 ± 108.7		65.1 ± 70.1		136.2 ± 142.7		192.7 ± 150.3		F_3,274_ = 10.69	<0.0001

All values are displayed as percentage and absolute number within the columns if not stated otherwise.

All differences did not reach statistically significance except *.

**ANOVA was conducted using the transformed D 25-OH and D3 1,25-OH values, respectively.

Values for “from nursing home” and “30 day mortality” were too small for statistical analysis.

### Correlation of 25-OH and 1,25-OH2 serum concentrations

**Figure 1:**
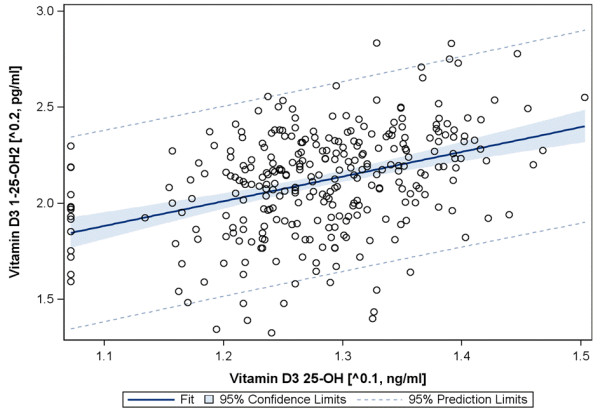
Correlation between 25-OH and 1,25-OH2 blood levels (raw data).

There was a modest positive correlation between 25-0H and 1,25-OH2 (Spearman Correlation Coefficient 0.38, p <0.0001; Figure 1). We found no relationship with CRP, neither for Vitamin D 25-OH nor for Vitamin D1,25-OH2.

### Impact of age and season

**Figure 2:**
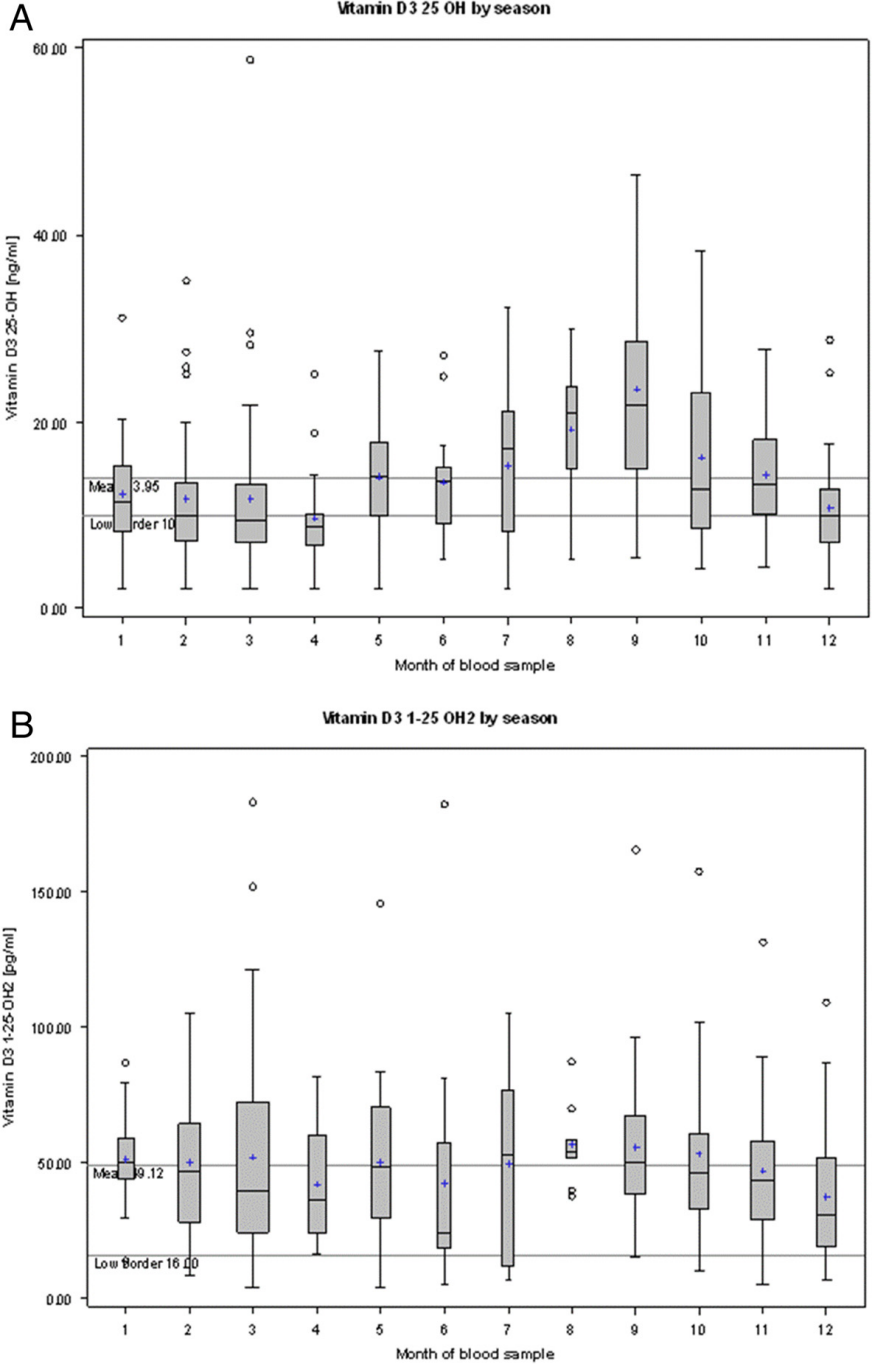
25-OH (A) and 1,25-OH2 (B) blood levels by season (raw data).

The well-known seasonal pattern of vitamin D metabolites was confirmed for 25-OH but not for 1,25-OH2 (F_11,287_ = 5.13, p < 0.001 and F_11, 287_ = 1.4, p = 0.172, respectively, based on power-transformed values; Figure 2). After seasonal adjustment, age had a significant impact on 1,25-OH2 levels (F_1,297_ = 6.06, p = 0.0144) but not on 25-OH levels (F_1,297_ = 0.03, p = 0.8588).

To control for the impact of season and age, all further analyses were performed based on seasonally and age adjusted D 25-OH and D3 1-25-OH2 values.

### Impact of pathogen

After adjustment for age and season, no significant differences between the four groups (pneumonia with no pathogen detected, pneumococcal pneumonia, legionella pneumonia, influenza pneumonia, Table 1) were detected. Surprisingly, there was a significant difference within the influenza group: When we separately analyzed influenza A (n = 36) and B (n = 14), we found equal to lower values for 25-OH in Influenza A compared to influenza B (10.8 ± 7.7 vs. 12.7 ± 13.8, F_1,48_ = 0.20, p = 0.6595) but significantly higher values for 1,25-OH2 in influenza A compared to influenza B (63.5 ± 38.3 vs 31.4 ± 24.1, F_1,48_ = 8.05, p = 0.0067).

### Impact of comorbidity

There was a general trend towards lower 25-OH and 1,25-OH2 values in presence of any co-morbidity (Table 2). However, only three associations revealed statistical significance: Liver disease was associated with significantly lower D 25-OH values (8.9 vs. 14.1 ng/ml, F_1,295_ = 4,84, p = 0.0285), respiratory disease was associated with significant lower D 25-OH levels (12.6 vs. 14.6 ng/ml, F_1,290_ = 4.70, p = 0.031) and renal co-morbidity was associated with lower D 1,25-OH2 values (34.1 vs. 50.2 pg/ml, F_1,295_ = 10.63, p = 0.0012).

**Table 2:** Impact of the presence of co-morbidity according to organ on 1,25-OH2 and 25-OH serum concentrations (transformed age and season adjusted values, bold numbers represent significant differences)

**Co-morbidity**			**1,25-OH in pg/ml**		**Statistics**	**25-OH in ng/ml**		**Statistics**
		**n**	**Mean**	**SD**	**p value**	**Mean**	**SD**	**p value**
Respiratory	No	199	50.7	32.3	F_1,296_ = 1.41	**14.6**	8.6	F_1,296_ = 4.70
	Yes	99	45.8	23.0	0.2356	**12.6**	8.3	0.0310
Hepatic	No	288	49.6	29.5	F_1,297_ = 2.09	**14.1**	8.5	F_1,297_ = 4.84
	Yes	11	37.2	27.9	0.1494	**8.9**	5.2	0.0285
Renal	No	**279**	**50.2**	29.7	F_1,297_ = 10.63	14.0	8.6	F_1,297_ = 1.81
	Yes	**20**	**34.1**	21.9	0.0012	12.7	7.7	0.1792
Diabetes mellitus	No	256	49.1	30.0	F_1,297_ = 0.41	14.1	8.8	F_1,297_ = 0.28
	Yes	43	48.9	27.2	0.5203	13.0	6.4	0.5945
Cardiac	No	260	49.9	30.1	F_1,296_ = 2.50	14.1	8.7	F_1,296_ = 1.41
	Yes	38	44.1	25.2	0.1151	13.2	6.8	0.2364

### Impact of CURB-score, hospitalization rate and duration

Because of the impact of age on 25-OH and 1,25-OH2 serum concentrations, we used the CURB instead of the CRB-65 score to investigate the association between initial severity of pneumonia and vitamin D. For 1,25-OH2 but not 25-OH, we found a significantly negative correlation with increasing CURB-score values. The general linear model revealed a significant association between low 1,25-OH2 serum concentration and high CURB scores (p = 0.011), whereas there was no significant association between 25-OH and CURB (p = 0.325).

Both, 25-OH and 1,25-OH2 were lower for patients who needed to be hospitalized: 12.8 ± 7.8 vs. 16.2 ± 10.0 ng/ml and 44.3 ± 28.2 vs. 58.2 ± 30.0 pg/ml, respectively.

We also found a significant impact of 25-OH and 1,25-OH2 serum concentrations -all samples were taken on admission- on duration of hospitalization. Lower levels were associated with longer hospitalization (F_1,290_ = 17.69 p < 0.001 and F_1,290_ = 46.16, p < 0.001, respectively).

There was no significant impact of neither 25-OH nor 1,25-OH2 serum concentrations on fatal outcome (Chi^2^_1_ = 0.2374, p = 0.6261 and Chi^2^_1_ = 0.1964, p = 0.6576 respectively). However, the number of deaths in this cohort was limited (n = 13). Since only 2 patients received mechanical ventilation, we did not analyse the impact on respiratory failure.

## Discussion

We found that 1,25OH2 serum concentration is inversely correlated with severity of CAP in means of the CURB score without any significant association to specific pathogens.

However, CURB score has been evaluated as predictor of mortality and is therefore only a surrogate parameter for severity. Admission to ICU as parameter for severity could not be accessed due to a large amount of missing data. Another limitation of our study is the lack of a healthy control group. Regarding ethnicity and geographical latitude, our cohort is comparable to the subgroup of non-Hispanic whites analyzed within the US- National Health and Nutrition Examination Survey (http://www.cdc.gov/nutritionreport/99-02/pdf/nr_ch2b.pdf). In this survey, approximately 10 percent of the general population had 25-OH concentrations < 11 ng/ml. In our cohort, 45.4% of the patients had 25-OH values of less than 11 ng/ml. The geometric mean of 25-OH for (not acutely ill) non-Hispanic whites in this survey was 21.6 ng/mL for adults between 20–59 years and 21.0 ng/mL for adults over 59 years, which is markedly higher than the values measured in our pneumonia cohort.

As others, we found a high variability of vitamin D metabolites with season and age accounting for the majority of the observed variances. Therefore, we decided to use a model controlling for the influence of season and age instead of sequential univariate-multivariate testing because it is well-known that these 2 factors have a substantial influence on vitamin D levels [9]. We did not detect significantly different transformed 1,25-OH2 and 25-OH levels between different respiratory pathogens, that probably provoke different pathways of the innate immune system. However, if we applied the cut off values for “deficiency”, “insufficiency” and “sufficiency” based on untransformed data, we found that the proportion of deficient patients was highest in the group of influenza pneumonia and lowest in the group of legionella pneumonia. This is likely to be explained by the fact that incidence of influenza and pneumococcal but not of legionella infections is increased during winter season.

The interpretation of the observed difference within the influenza group between influenza A and B is difficult. Because vitamin D skews the immune system towards a more tolerogenic state, some authors have hypothized that a high vitamin D serum levels might result in a lower antibody response to influenza infection and vaccination [10-12]. However, this was not confirmed in a recent trial on vaccinated children that were supplemented with 1000 IU of vitamin D [13]. The differences within the influenza subgroups observed in our cohort may be generated simply by chance, because the investigated numbers in the subgroups were low.

When we investigated the association of vitamin D with severity, we found that lower 1,25-OH but not 25-OH levels were significantly associated with increased CURB score. For 25-OH, we detected only a non-significant trend. A recent study on 272 Dutch CAP patients identified 25-OH deficiency (same cut off as in our study) as independent predictor for ICU admission and mortality. However, the authors did neither control for impact of age and season nor measure 1,25-OH2 [14]. Controlling for season is of major importance, since most cases of CAP occur during winter, when vitamin D levels, particularly 25-OH, are lower in the general population. In that study, 53% of patients exhibited deficiency compared to 82% in our study. The 180 days mortality was similar with 5.8% (our study 4.3%).

In our study, low 1,25-OH was significantly associated with renal co-morbidity but not hepatic co-morbidity, whereas low D 25-OH levels were significantly associated with hepatic and pulmonary but not renal-comorbidity. All these statistically significant associations can be explained by the vitamin D metabolism: the 25-hydroxylation occurs in the liver and the consecutive 1-hydroxylation in the kidney. Obviously, low 25-OH levels can be compensated for by increased renal hydroxylation. Vice versa, sufficient 25-OH levels do not necessarily translate into sufficient 1,25-OH2 levels. The latter is supported by the admittedly significant but non-marked correlation between those two metabolites.

Altogether, this may provide a possible explanation for the contradictory results of interventional studies on supplementation if vitamin D. Obviously, supplementation of vitamin D is of minor benefit, if it cannot be transformed into 1,25-OH2. This may explain that supplementation may prevent respiratory tract infections in otherwise healthy children [6] but not in elderly adults with co-morbidities [15], and it may also add explanation for the well-known association between renal co-morbidity and pneumococcal disease [7].

Similar to us, a recent study in septic patients found no significant difference for 25-OH but for 1,25-OH2 blood levels between survivors and non-survivors at baseline. They describe that for the same level of 25-OH, 1,25-OH2 would be lower in non-survivors compared to survivors [16]. The authors measured also the 24-hydroxylase activity and concluded that the decreased 1,25-OH in the non-survivors points to a decreased production and not to increased degradation.

Therefore, to measure only the reservoir form (25-OH) -as recommended by guidelines to determine the vitamin D “status”- maybe insufficient. Effects of vitamin D are primarily conferred by the active form (1,25-OH2) and there is no linear relationship between the serum levels of these 2 metabolites. In line with our approach, Powe et al. stressed the importance of studying not only 25-OH but also 1,25-OH2 to understand the complex effects of vitamin D metabolism in a recent study published in the New England Journal of Medicine [17,18].

As a consequence, supplementation of 1,25-OH instead of vitamin D may be more appropriate to exploit potential protective effects of vitamin D, particularly in elderly persons with co-morbidities, who exhibit an increased risk for pneumonia. While animal sepsis models investigating 1,25-OH2 administration have shown promise, there is no human randomized clinical trial [19-21]. Supplementation with 1,25-OH2 is already the standard of care for patients depending on hemodialysis to prevent osteoporosis and hyperparathyroidism. However, 1,25-OH2 imposes a greater risk for toxicity and demandable clinical trials would therefore need a thorough monitoring of 1,25-OH2 levels.

## Conclusion

According to our results, the link between vitamin D deficiency and increased susceptibility to respiratory tract infections is rather conferred by the decreased levels of the activated form (1,25-OH2) than by decreased levels of the vitamin D reservoir form (25-OH), that is naturally lower in the winter-pneumonia season. Using transformed data of 25-OH and 1,25-OH2 to effectively control for age and season, we found a significant and independent negative correlation between low 1,25-OH2 levels and pneumonia severity, which could not be detected for 25-OH. Our findings may explain, why vitamin D supplementation targeting to normalize 25-OH levels alone in a cohort with decreased capacity (e.g. because of age and co-morbidities) for activation of vitamin D may not be sufficient to exploit the suspected protective effects.

## Competing interests

The authors declare that they have no competing interests.

## Authors’ contribution

MWP, CT, TW and RB designed the study. CT preformed the vitamin D measurement. MWP drafted the manuscript. US conducted the statistical analysis. All authors contributed to writing and interpretation of data. All authors read and approved the final manuscript.
